# Draft Genome of Chilean Honeybee (*Apis mellifera*) Gut Strain *Lactobacillus kunkeei* MP2

**DOI:** 10.1128/genomeA.01013-14

**Published:** 2014-10-09

**Authors:** Alejandro Olmos, Patricia Henríquez-Piskulich, Carolina Sanchez, Marcelo Rojas-Herrera, Mario Moreno-Pino, Marcela Gómez, Rafael Rodríguez Da Silva, Vinicius Maracaja-Coutinho, Patricia Aldea, Annette N. Trombert

**Affiliations:** a Centro de Genómica y Bioinformática (CGyB), Facultad de Ciencias, Universidad Mayor, Santiago, Chile; b Ceapi Mayor, Universidad Mayor, Santiago, Chile

## Abstract

Here, we report the first draft genome sequence of *Lactobacillus kunkeei* strain MP2, isolated from a Chilean honeybee gut. The sequenced genome has a total size of 1.58 Mb distributed into 44 contigs and 1,356 protein-coding sequences.

## GENOME ANNOUNCEMENT

Honeybee (*Apis mellifera*) is the most important pollinator in worldwide agriculture, playing a key role in the human food supply (1). However, since 2006 an unusual decrease in honeybee colonies has been taking place. Thus, it is imperative to find alternative strategies to promote the health in beehives. In addition, the presence of commensal bacteria in *Drosophila* is directly associated with insect health status (2, 3). Therefore, the knowledge of honeybee microbiota members may contribute to an understanding of the symbiont-host interaction and its relation to insect health (4, 5). *Lactobacillus kunkeei* (*L. kunkeei*) is a honeybee microbiota bacterium associated mainly with the crops (6) and gut (7).

Genomic DNA of *L. kunkeei* MP2 was fragmented with NEBNext dsDNA Fragmentase (New England Biolabs, Ipswich, MA) and used to prepare a paired-end library. The library was sequenced using a MiSeq sequencer (Illumina, San Diego, CA) at the Genomics and Bioinformatics Center (CGyB, Universidad Mayor, Chile).

*De novo* assembly of the reads was performed using the software MaSuRCA version 2.0.3.1 (8). A total of 1,581,395 bp in 44 contigs with an average length of 34,511 bp (largest contig, 156,867 bp; shortest contig, 302 bp) are part of the draft genome sequence of *L. kunkeei* MP2. Additionally, a total of 96,293 bp of *N*_50_, a G+C content of 36.7%, and mean coverage of 10× were observed.

Prediction and annotation of protein coding DNA sequences (CDSs) and ribosomal genes (tRNAs, rRNAs, and ncRNAs) were performed using the NCBI Prokaryotic Genomes Automatic Annotation Pipeline (PGAAP version 2.6). Applying this pipeline we were able to identify 1,446 coding and noncoding genes. Of these, 1,356 are CDSs, 7 pseudo-genes, 18 rRNAs, 64 tRNAs, and 1 ncRNA. Finally, three features were classified as potential frameshifted genes. Exploring the structural rRNAs genes predicted by PGAAP, we identified 7 16S, 6 23S, and 5 5S ribosomal RNAs. Only one 16S and one 23S are completely represented, containing a total length of 1,662 bp and 2,920 bp, respectively.

In the context of coding sequences annotated in this first version, we identified 826 well-annotated protein-coding genes, and 530 annotated as hypothetical proteins. We found a chloramphenicol acetyltransferase (CAT), an enzyme responsible of chloramphenicol resistance. When the enzyme is used as a veterinary drug, chloramphenicol residues can be found in honey (9, 10). Also, we found 17 transposases, suggesting the possibility of a large diversity of transposable elements (11). These genes in *L. kunkeei* suggest that the strain has the potential to adaptation to different environmental conditions.

This draft sequence provides a new repertoire of genes and genome information for this strain of the symbiotic-host bacterium *Lactobacillus kunkeei*. It can be useful for future studies on the roles of microbiota in honeybee.

### Nucleotide sequence accession numbers.

This whole-genome shotgun project has been deposited at DDBJ/EMBL/GenBank under the accession number JPUI00000000. The version described in this paper is version JPUI01000000.
